# Molecular Characterization of Persistent SARS-CoV-2 Infections in Immunocompromised Patients

**DOI:** 10.3390/v18020189

**Published:** 2026-01-30

**Authors:** Patricia Volkow-Fernández, Marco Villanueva-Reza, Santiago Ávila-Ríos, Enrique Mendoza-Ramírez, América Citlali Vera-Jimenez, Alexandra Martin-Onraet, Beda Islas-Muñoz, Pamela Alatorre-Fernández, Rogelio Pérez-Padilla, Daniel Carpio-Guadarrama, Andrea Cárdenas-Ortega, Víctor Hugo Ahumada-Topete, Clara Espitia, Karen Lizbeth Reyes-Barrera, Edgar Sevilla-Reyes, Joel Armando Vázquez-Pérez

**Affiliations:** 1 Department of Infectious Diseases, Instituto Nacional de Cancerología, Mexico City 14080, Mexico; pvolkowf@gmail.com (P.V.-F.); alexitemaon@gmail.com (A.M.-O.); bedaislas@gmail.com (B.I.-M.); pamelaalatorre@hotmail.com (P.A.-F.); daniel.carps.81@gmail.com (D.C.-G.); 2 Hospital Epidemiology and Infectology Department, Instituto Nacional Enfermedades Respiratorias Ismael Cosio Villegas, Mexico City 14080, Mexico; marco.vilre@gmail.com (M.V.-R.); victor.ahumada@uehi.mx (V.H.A.-T.); 3 Centro de Investigación en Enfermedades Infecciosas, Instituto Nacional Enfermedades Respiratorias Ismael Cosio Villegas, Mexico City 14080, Mexico; santiago.avila@cieni.org.mx (S.Á.-R.); andrea.cardenas@cieni.org.mx (A.C.-O.); 4 Laboratory of Molecular Biology of Emergent Infectious Disease, Instituto Nacional Enfermedades Respiratorias Ismael Cosio Villegas, Mexico City 14080, Mexico; heinrichunam@gmail.com (E.M.-R.); americacitlali.vera52@gmail.com (A.C.V.-J.); 5 Department of Research in Tobacco and COPD, Instituto Nacional Enfermedades Respiratorias Ismael Cosio Villegas, Mexico City 14080, Mexico; perezpad@gmail.com; 6 Department of Immunology, Institute of Biomedical Research, Universidad Nacional Autónoma de México (UNAM), Mexico City 04510, Mexicokarenreyesbarrera@iibiomedicas.unam.mx (K.L.R.-B.); 7 Laboratory of Transcriptomics and Molecular Immunology, Instituto Nacional Enfermedades Respiratorias Ismael Cosio Villegas, Mexico City 14080, Mexico

**Keywords:** SARS-CoV-2, persistent infection, immunosuppression

## Abstract

Immunocompromised patients, including those with advanced HIV infection, hematologic malignancies treated with anti-CD20 monoclonal antibodies, or combined immunodeficiencies, are at increased risk of persistent SARS-CoV-2 infection. While long-term viral shedding has been described in these patients, the extent and nature of intra-host viral evolution during long-term infection remain insufficiently documented. In this study, we report longitudinal genomic analyses of SARS-CoV-2 from three immunocompromised individuals with persistent COVID-19: (i) a female patient with follicular lymphoma receiving bendamustine-rituximab therapy with 9 months of persistence, (ii) a male patient with advanced HIV infection following prolonged antiretroviral therapy interruption with 10 months of persistence, and (iii) a female patient with Good’s Syndrome characterized by combined humoral and cellular immune deficiency with apparently four years of persistence. Replication-competent virus was detected over extended periods. Sequential whole-genome sequencing revealed the gradual accumulation of non-synonymous mutations across multiple viral genes, consistent with ongoing viral replication and intra-host diversification in the absence of effective immune control. Although based on a limited number of cases, these findings provide descriptive evidence that persistent SARS-CoV-2 infection in immunocompromised hosts can be associated with sustained viral evolution. This work highlights the importance of continued virological monitoring in selected patients with prolonged infection and contributes to the understanding of SARS-CoV-2 dynamics in settings of impaired immunity.

## 1. Introduction

The COVID-19 pandemic posed a significant challenge to global public health. Vaccination and, when accessible, monoclonal antibodies and antivirals against SARS-CoV-2 have substantially reduced morbidity and mortality; however, immunosuppressed patients continue to be a major concern as they have higher morbidity and mortality due to COVID-19 [1]. These patients with impaired immunity, due to the disease itself or therapy, have a lack of adequate vaccination response, and also, these individuals might prolong viral shedding, which may contribute to the emergence of new variants [2].

One of the most recognized risks associated with prolonged COVID disease is having received anti-CD20 therapy with rituximab, and the shorter the period before infection or during infection, the worse the outcomes [3]. Patients receiving immunotherapy with anti-CD20 face enormous challenges as they have almost no response to vaccines [4], and once infected, the risk of adverse outcomes, prolonged course, or relapsing disease has also been reported. Moreover, prolonged shedding of SARS-CoV-2 has been observed in patients with impaired humoral immunity, including adults and children [5]. Patients with different primary or secondary immunodeficiencies may differ in the extent of viral shedding, viral replication kinetics, and disease severity [6].

Humoral immunity has been recognized as a significant determinant in resolving SARS-CoV-2 infection. Patients with B-cell function impairment often mount almost no antibody production against SARS-CoV-2 infection and have been reported to benefit from convalescent plasma to resolve prolonged SARS-CoV-2 shedding [5]. Chronic replication of SARS-CoV-2 in these patients has important consequences, as the loss of immunological selection pressure increases viral diversity with the occurrence of mutations throughout the genome [7].

Persistent or prolonged COVID-19 infection refers to ongoing viral RNA replication, which has been reported with varying durations. Defining and managing persistent COVID-19 remains complex. While some argue against treatment, others propose treatment of patients with persistent viral replication to prevent the possible emergence of new variants and halt the chain of transmission. Persistent COVID-19 is thought to be more common in individuals with humoral deficiencies, but it has also been reported in people with HIV and those who have undergone solid organ transplantation. Additionally, some patients may continue to shed the virus while remaining minimally symptomatic or asymptomatic, further complicating decision-making.

Persistent infections have been proposed as potential sources of highly divergent viral lineages that may give rise to variants of concern (VOCs), such as Alpha or Omicron. For this reason, the study of immunocompromised patients has been of importance as a possible source of variants [8]. Substitutions in RBD Spike positions 452, 478, 484 and 501 and deletions H69/70 and Y144 in N-terminal domain are signatures of VOCs and their frequent detection in immunocompromised patients supports the hypothesis regarding the origin of novel variants [9]. While mutations associated with immune evasion and host immunity play a significant role in viral persistence, the underlying mechanisms still need to be fully understood. Here, we describe three clinical cases of prolonged COVID-19: one patient with follicular lymphoma treated with rituximab, one person living with HIV, and one patient with thymoma. The molecular characterization of the complete SARS-CoV-2 genome longitudinally and the phylogenetic and viral mutations analyses showed a distinct evolutionary pattern in three immunocompromised patients.

## 2. Methodology

### 2.1. SARS-CoV-2 Diagnostics

Oropharyngeal or nasopharyngeal swabs were collected, and the diagnosis was made using validated RT-qPCR protocols for SARS-CoV-2 RNA detection, approved by the World Health Organization (WHO).

### 2.2. RNA Extraction and Sequencing

Viral nucleic acid extraction was performed using QIAamp viral RNA Minikit (Qiagen, Hilden, Germany). Libraries for SARS-CoV-2 sequencing were made according to the ARTIC Lo-Cost protocol, V4.1 primers. High-accuracy base calling was performed using Guppy (v4.4.0) (Oxford Nanopore Technologies, Oxford, UK) and demultiplexed using the Guppy barcoder. Downstream analysis was performed using the epi2me pipeline, including Fasta QC (v0.12.x), wf-ARTIC (v1.2.2), and NextClade (v3.18.1) software, to generate a consensus sequence for each sample in FASTA format.

### 2.3. Phylogenetic Analysis

To perform phylogenetic analysis, we included the obtained complete sequences (*n* = 6) and selected 234 virus genomes available on the GISAID platform (https://gisaid.org, accessed on 1 June 2025). Sequence alignments were created with MAFFT V7 [10] and edited with MEGA 10.0 [11]. A maximum likelihood tree was constructed for the whole Spike sequence using MEGA 10.0. The General Time-Reversible (GTR) model was selected with five-parameter gamma-distributed rates and 1000 bootstrap replicates. The edition of the trees was made using FigTree v1.4.4 (http://tree.bio.ed.ac.uk/software/figtree/ (accessed on 13 March 2024) [12].

### 2.4. Detection of Genomic (gE RNA) and Subgenomic E RNA (sgE RNA)

We used a PCR design described previously to detect sgE RNA (plus strand). We used a leader-specific primer described previously [13], sgLeadSARSCoV2-F (CGATCTCTTGTAGATCTGTTCTC), and the primers and probes targeting sequences downstream of the start codons of the E gene (gE RNA) described previously. To validate the detection of sgE RNA, a positive signal must be obtained for both gE RNA and the human gene (RNAse P). sgE RNA detection was used as a surrogate marker of ongoing viral replication.

### 2.5. SARS-CoV-2 Isolation from Respiratory Sample, Patient 2

Viral isolation was performed from a nasopharyngeal swab from day 189 to confirm the presence of replication-competent virus. The sample was centrifuged at 3000 rpm, and the supernatant was collected and filtered using a 0.22 µm membrane. Dilution was performed with Minimum Essential Medium Eagle (EMEM) medium (Sigma-Aldrich, St. Louis, MO, USA) and 2% Fetal Bovine Serum (Gibco™ Grand Island, New York, NY, USA). A total of 600 μL of the diluted sample was placed in a 25 cc culture flask with confluent Vero E6 cells (ATCC Cat. No.CRL-1586). Incubation for 90 min at 37 °C was performed, and 5 mL of EMEM + 2% FBS medium was added, incubating at 37 ° C for 72 h. Supernatant was harvested and clarified by centrifugation at 3000 rpm for 5 min in the flask with a cytopathic effect. Viral RNA was obtained using QIAamp viral RNA Minikit (Qiagen, Hilden, Germany) and sequenced in order to confirm the identity of isolate.

### 2.6. Patient Consent

All patients signed an informed consent form for their samples to be analyzed and their cases studied for research purposes. Protocol INER B22-23 was approved by the Institutional Research Committees of the Instituto Nacional de Enfermedades Respiratorias.

## 3. Results

### 3.1. Clinical Course and Virus Evolution

For this report, patients’ records and laboratory data were integrated. In all three cases, persistent SARS-CoV-2 infection was defined by prolonged RT-qPCR positivity (months), which is supposed to be the product of sustained viral genomic lineage replication and monophyletic clustering over time; distinguishing these cases from post-acute COVID-19 sequelae (long COVID), which typically occur in the absence of ongoing viral replication. However, despite viral persistence in all three patients, the extent and pattern of viral evolution differed and was influenced by the nature of the immune deficiency of each patient.

#### 3.1.1. Case 1

**Figure 1:**
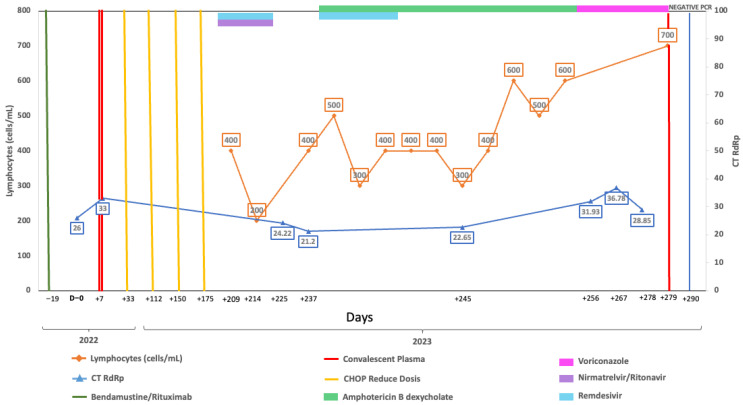
Longitudinal virological and immunological timeline of Case 1. Timeline showing key therapeutic interventions, lymphocyte counts, and SARS-CoV-2 RT-qPCR cycle threshold (Ct) values during follow-up. The X-axis represents dates, the left Y-axis indicates absolute lymphocyte count (cells/µL), and the right Y-axis indicates RT-qPCR Ct values targeting the RdRp gene. Solid lines depict longitudinal measurements of lymphocyte counts and Ct values. Top horizontal bars indicate periods of antiviral or immunochemotherapy treatment. Vertical lines mark the timing of convalescent plasma or CHOP administration. Vertical blue line indicates the time of RT-qPCR negative result.

A 58-year-old woman, vaccinated with a single dose of BNT162b2 in 2021, was first admitted on 19 May 2022, with follicular lymphoma Grade 3a Stage IV FLIPI Score 3. Chemotherapy with bendamustine-rituximab began on 8 June 2022, and she completed four cycles by 1 September 2022 (Figure 1). On 20 September she was admitted to the emergency department with respiratory symptoms, including a 6-day history of sore throat, productive cough, and hypoxemia (her pulse oximeter at home showed 80%). A rapid antigen test for SARS-CoV-2 was positive, confirming severe COVID-19, and she was treated with remdesivir for 5 days, supplementary oxygen, and dexamethasone. She was discharged on 25 September 2022, but her RT-qPCR test still was positive and had negative neutralizing antibodies. Due to the risk associated with chemotherapy, convalescent plasma was considered and administered. Chemotherapy was resumed with R-CHOP (4 cycles), completing treatment by 14 March 2023. On 17 April, a new episode of shortness of breath with a non-productive cough and hypoxemia brought the patient to the emergency department. SARS-CoV-2 was detected through RT-qPCR, confirming severe COVID-19, and combination treatment with remdesivir and nirmatrelvir/ritonavir was given for 5 days. Severe was considered based upon hypoxemia. A CT scan on 21 April was relevant for bilateral diffuse interstitial ground glass opacities. After completing the antiviral therapy, she was discharged on 23 April 2023. A RT-qPCR was taken prior to starting antiviral treatment.

Despite antiviral treatment, the patient had a positive antigen and RT-qPCR test at a follow-up visit on 3 May 2023 while remaining symptomatic. A follow-up CT scan on 10 May showed no important changes in ground glass opacities. Due to oxygen requirements and symptomatology, she was hospitalized on 17 May. Upon admission, RT-qPCR continued to be positive considering now-persistent COVID-19 rather than new infection. Additional tests, including neutralizing antibodies, continued to be negative, while RT-qPCR tests performed on 2, 14, and 27 June were positive. Due to her immune status and lack of improvement, a second dose of convalescent plasma to prevent progression was administered with a first negative RT-qPCR test on 7 July.

**Figure 2:**
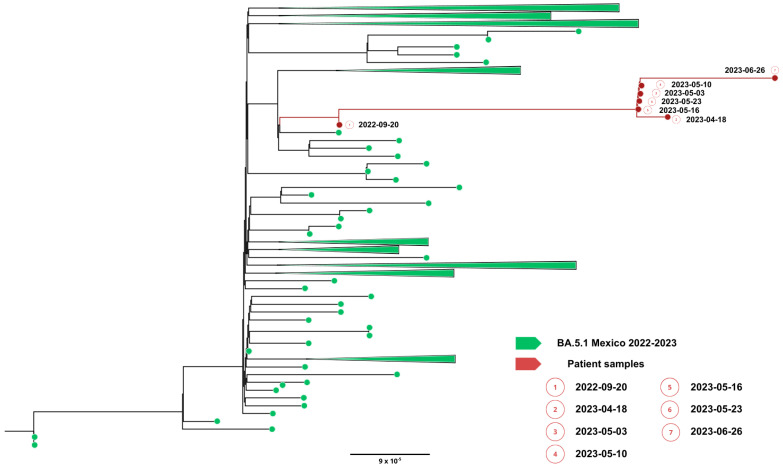
Phylogenetic evidence of persistent SARS-CoV-2 infection in Case 1. Maximum likelihood phylogenetic tree constructed from whole-genome consensus SARS-CoV-2 sequences, including seven longitudinal samples obtained from Case 1 (red tips) and 234 contemporaneous sequences of the Omicron sublineage BA.5.1.23 circulating in Mexico during the same period. Case 1-derived sequences form a monophyletic cluster, consistent with persistent infection and intra-host viral evolution rather than reinfection. Bootstrap support values (>90%, 1000 replicates) support the clustering of patient-derived sequences. The scale bar indicates nucleotide substitutions per site.

Complete viral genome sequences from nasopharyngeal swabs obtained on September 20, 2022 (day 0), 16 April (day 208), 2 May (day 224 and 30 days after remdesivir with nirmatrelvir/ritonavir), 5 May (day 227), 9 May (day 231), 22 May (day 244), 25 May (day 247) (GISAID accession IDs EPI_ISL_15288392, EPI_ISL_17960444, EPI_ISL_17960440, EPI_ISL_17960441, EPI_ISL_17960442, EPI_ISL_17960443 and EPI_ISL_18059424) and 26 June (day 277) showed a SARS-CoV-2 infection with a unique SARS-CoV-2 Omicron subvariant BA.5.1.23, ruling out reinfection with other omicron subvariants, persistent for more than 9 months. Phylogenetic analysis based on the whole genome showed that viral genomes of the patient formed a robust monophyletic group (Figure 2); the root of the cluster was created by the first sequence from a sample obtained in September 2022, and the subsequent samples originated from this sample, consistent with persistent infection and intra-patient viral evolution (Figure 2). Non-synonymous mutations detected in the second sample were observed within the M (S197N, A38S), ORF8 (Del 86–88), ORF9b (R13L), and predominantly in the ORF1ab coding sequences (L2146F, H2357Y, R3802H, V3917G, T4175I) when compared to the Wuhan sequence, seven months after the first SARS-CoV-2 positive sample. Because the patient was asymptomatic between these samples, we could not document the time of the mutations’ appearance. No emergent mutations in antigenic regions or RBD of the Spike protein during the nine months of persistence were detected. The absence of Spike RBD mutations despite persistent infection suggests limited humoral immune pressure, consistent with profound B-cell depletion.

**Figure 3:**
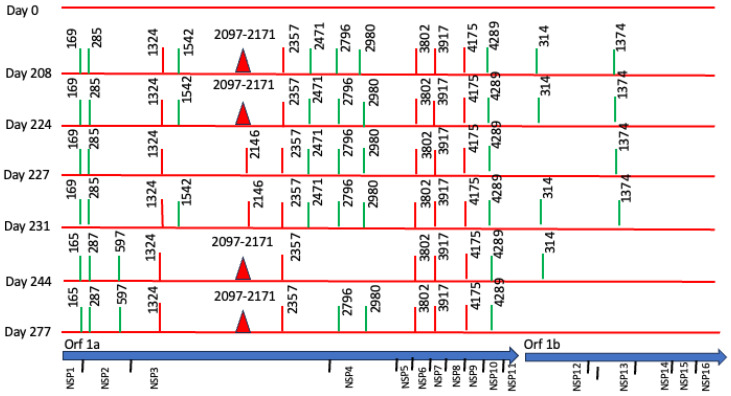
Longitudinal Case 1 intra-host SARS-CoV-2 genomic evolution. Schematic representation of consensus-level mutations and polymorphisms identified across encoded non-structural proteins in seven longitudinal SARS-CoV-2 genome sequences obtained from Case 1 between day 0 and day 277. Amino acid substitutions relative to the Wuhan reference genome are shown for all non-synonymous changes (red), synonymous changes (green), and deletions (closed red triangles). Some substitutions (e.g., amino acids 165, 1324, 2357, 3802, 3917, 4175, 4289) persisted across multiple time points, consistent with fixation during prolonged infection. For context, the lower panel shows the genomic organization of SARS-CoV-2.

However, we found non-synonymous polymorphisms in ORF1ab that were fixated from the second sample (Table 1, Figure 3). We also detected deletions in Orf1a and N proteins. These findings point out the presence of viral quasispecies during the persistence of SARS-CoV-2.

Persistent replication was characterized by subgenomic detection. Genomic viral E RNA (gE RNA) and subgenomic E RNA (sgE RNA) were detected in all samples collected on days 0, 228, 224, 227, 231, 244, and 277 after the initial diagnosis, as an indication of persistent viral replication. The values of gE CTs (median = 24.94, R = 21.10–28.60) were consistently lower compared with sgE RNA (median = 31.53, R = 27.88–35.13).

#### 3.1.2. Case 2

**Figure 4:**
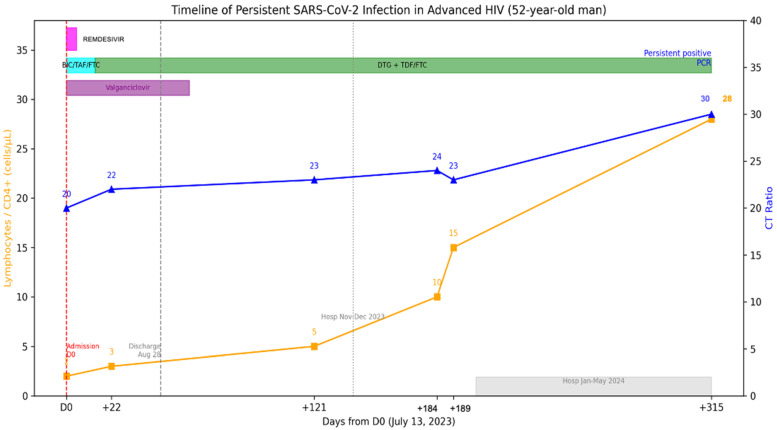
Longitudinal virological and immunological timeline of Case 2. Patient with advanced HIV (CD4 2–28/µL), persistent RT-qPCR positivity for >8 months despite ART, finally cleared after immune reconstitution. The X-axis represents dates, the left Y-axis indicates percent lymphocyte count (cells/µL), and the right Y-axis indicates RT-qPCR Ct values. Solid lines depict longitudinal measurements of lymphocyte counts and Ct values. Top horizontal bars indicate periods of antiviral treatment.

A 52-year-old man was admitted on 13 July 2023 to the emergency department with chronic headache and visual loss, accompanied by weight loss, cough, dyspnea, dysphagia, and diarrhea. The patient’s prior medical history is relevant for HIV diagnosed in 2002 without antiretrovirals since 2018 and loss of follow-up. HIV viral load was on 5.56 log copies/mL, and CD4+ T-cell count was 2 cells/uL (2%) (Figure 4). He reported having completed three doses COVID-19 vaccination and no prior COVID-19.

The RT-qPCR test was positive for SARS-CoV-2, and severe COVID-19 was diagnosed, for which remdesivir was administered upon admission. A CT scan showed subpleural micronodules, ground glass patches, and bronchiectasis. BAL and lung biopsy through bronchoscopy were done with cultures positive for *K. oxytoca* and *S. pneumoniae*. Other opportunistic infections, including Mycobacterium avium (MAC) and GI and SNC Cytomegalovirus (CMV) were diagnosed. Thus, valganciclovir was started. Combination treatment was initially started with bictegravir, tenofovir alafenamide and emtricitabine (FTC); later, it was modified to Dolutegravir 50 mg BID, tenofovir disoproxil succinate (TDS) and FTC. The patient remained hospitalized due to opportunistic infections, and SARS-CoV-2 RT-qPCR was performed, and the patient continued to be positive; this test was 22 days after antiviral treatment. He was discharged on August 28, 2023 but was hospitalized in November due to problems with food intake leading to malnutrition and discharged in December 2023, and again in January until May 2024 for similar situations. During this period the patient had no respiratory symptoms, but the RT-qPCR remained positive for SARS-CoV-2. No further CT scans were performed. An additional positive test was detected on 22 May 2024. After initial antiviral treatment, no further antiviral treatment was administered, as the hospitalization was non-COVID related. Currently, he has an undetectable HIV viral load and CD4+ T-cell count of 28 (6%).

Mutations and polymorphisms detected in persistent SARS-CoV-2 and complete genome sequences were obtained from nasopharyngeal swabs collected on days 0 (13 July 2023), 22, 121, 181, 189 and 315 (22 May 2024) (GISAID accession ID, EPI_ISL_18415832, EPI_ISL_18415854, EPI_ISL_18798204, EPI_ISL_18798234, EPI_ISL_18969735 and EPI_ISL_19259365). All sequenced SARS-CoV-2 genomes from 13 July 2023 (day 0) to 22 May (day 315) had samples showing a unique omicron subvariant BA.2, confirming persistent infection.

**Figure 5:**
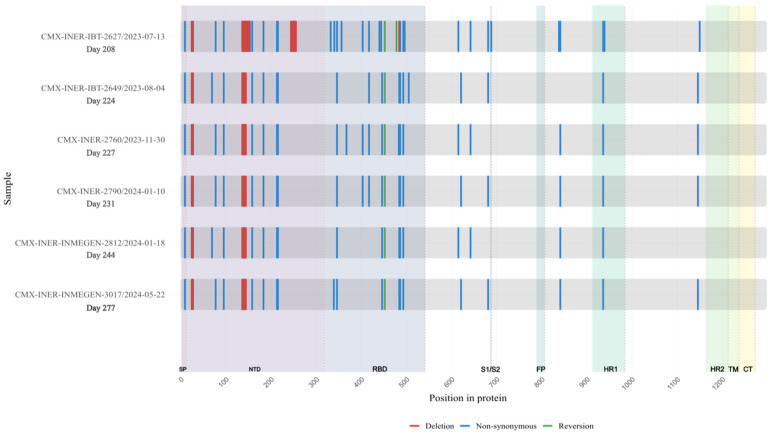
Longitudinal Case 2 accumulation of Spike protein mutations. Schematic representation of amino acid substitutions detected in the SARS-CoV-2 Spike protein across six longitudinal samples obtained from Case 2 during persistent infection. The X-axis indicates time (days since the first SARS-CoV-2–positive sample). Non-synonymous substitutions are shown in blue, and deletions are shown in red. The figure illustrates the temporal accumulation and persistence of Spike mutations during prolonged infection.

**Figure 6:**
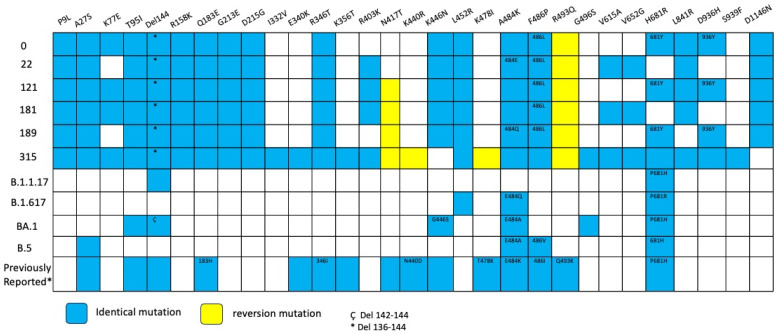
Overview of substitutions present in the Spike protein genome over the course of infection, Case 2. Summary of mutation site shared with variants of concerns such as alpha, delta, omicron BA.1, omicron BA.2 and previously mutations reported in persistent virus. Blue squares indicate the same amino acid position and number inside different amino acid change. Yellow squares indicate reversion mutation. * 136–144 del. ^Ç^ 142–144 del.

Extensive mutations were found in five genes (Orf1a, Orf3a, Orf9b, N and Spike) compared with BA.2 lineage (Table S1). Notably, Spike mutations were extremely convergent to highly mutated, chronic-infection sequences [14], R493Q reversion, R403K, P621S, A484K, and G446S (Figure 5, Table S2). When comparing with the lineage BA.2.86, we detected 17 Spike substitutions and one large deletion (∆138–144) that are in two or more of these sequences, including P9L, H69D, K77E, T95I, ∆136–144, R158K, Q183E, G213E, D215G, R346T, L452R, F486L, V615A, V642G, H681Y, L841R, D936Y, and D1146N different from the parent lineage. We detected four mutations that were shared between BA.2.86 and the six sequences of the patient: R403K, A484K, R493Q (reversion), and P621S (Figure 6). Moreover, we detected substitutions that are shared with persistence virus reported from other immunocompromised patients (Figure 6). The amino acid positions are 27, 95, 183, 215, 340, 346, 356, 417, 440, 446, 478, 484, 486, 490, 493, 501, 614, 681 and deletions in N-terminal domain 63–70 and 142–144.

**Figure 7:**
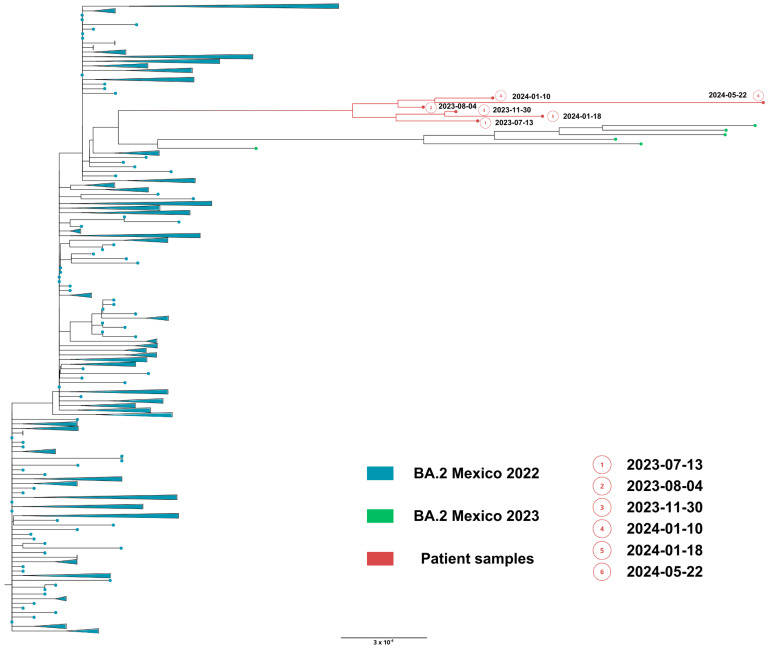
Maximum likelihood phylogenetic tree constructed from whole-genome consensus SARS-CoV-2 sequences, including six longitudinal samples obtained from Case 2 (red tips) and 234 contemporaneous sequences of lineage BA.2 from Mexico during 2022–2023. Case 2-derived sequences form a monophyletic cluster, consistent with persistent infection and intra-host viral evolution. Bootstrap values (>90%, 1000 replicates) support the clustering of patient-derived sequences. The scale bar indicates nucleotide substitutions per site.

Phylogenetic analysis based on the whole genome showed that viral genomes of the immunocompromised patient formed a robust monophyletic clade (Figure 7), consistent with persistent infection and intra-patient viral evolution. Patient sequences clustered with sequences circulating in Mexico in 2022 but were highly divergent to the rest of the isolates and patient sequences (Figure 7).

Infectious SARS-CoV-2 was successfully cultured from the nasopharyngeal swabs collected on day 189. The SARS-CoV-2 isolate showed a cytopathic effect on Vero E6 cells supporting persistent SARS-CoV-2 infection. The viral sequence was obtained from the cell culture (GISAID accession number ID EPI_ISL_20306021), confirming the identity of the isolated patient virus. However, comparison of the full-genome sequences obtained directly from the individual’s samples with the genome data obtained from the SARS-CoV-2 isolate showed point mutations in different genes: Orf1a: S2103F, V3349F, T4161I; Orf1b: A520V, P1548L, S:Q836R, Q992H, M:H125Y; Orf7b: H42Y, Orf8: R115C. The variation observed between the different genomes points to a quasispecies with continuous turnover of dominant viral species.

#### 3.1.3. Case 3

A 57-year-old woman with no prior pathological medical history presented with symptoms of upper and lower respiratory tract disease compatible with a mediastinal mass in February 2020. She sought medical attention in June when additional respiratory symptoms presented, and a COVID-19 PCR test was ordered that turned positive. A chest X-ray showed a mediastinal mass and pneumonia compatible with COVID-19. She was transferred to a third-level hospital as an outpatient. In June 2020, despite a SARS-CoV-2 RT-qPCR positive test, she was hospitalized and treated with dexamethasone and ivermectin, and upon completing treatment, she was discharged. RT-qPCR tests in July 2020 and August 2020 continued to be positive; nonetheless, a biopsy was performed, which revealed a thymoma. As resection was required, and the patient continued to be COVID-19 positive; she was discharged and with external follow-up until turning SARS-CoV-2 RT-qPCR negative.

**Figure 8:**
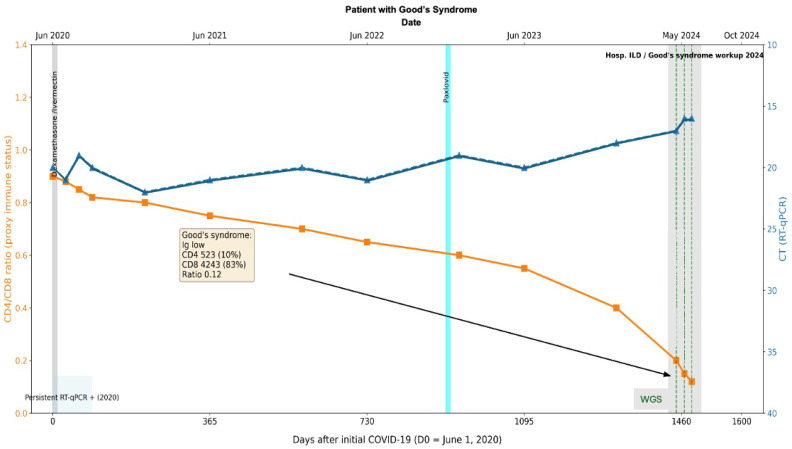
Longitudinal virological and immunological timeline of Case 2. Patient with thymoma and Good’s Syndrome (hypogammaglobulinemia, CD4/CD8 ratio 0.12), persistent SARS-CoV-2 infection >4 years with repeated RT-qPCR positivity. The X-axis represents dates, the left Y-axis indicates percent lymphocyte count (cells/µL), and the right Y-axis indicates RT-qPCR Ct values. Solid lines depict longitudinal measurements of lymphocyte counts and Ct values. Transversal bars indicate periods of antiviral treatment. The black arrow and box denote the timepoint of Goods syndrome diagnosis, with corresponding laboratory values in yellow box obtained at diagnosis.

In 2021, antigen tests for COVID-19 were positive. In 2023, while completing the workup for the thymoma, a SARS-CoV-2 RT-qPCR test was performed and came back positive (Figure 8). Initially considered an acute infection, the patient was given Paxlovid (Nirmatrelvir Ritonavir) without viral clearance. Further workup in April 2024 included a CT scan, which showed changes that were compatible with an interstitial pulmonary disease for which the patient was hospitalized. The immune status evaluation revealed: hypogammaglobulinemia, decreased CD4+ T-cell counts (523 absolute and 10%) and increased CD8 T-cells (4243 absolute and 83%) with a ratio of 0.12. The patient was considered to have a secondary immune deficiency compatible with Good’s Syndrome. COVID-19 PCR tests continued to be positive on 7 June 2024, 24 June 2024 (average CT 16), and 12 July 2024 (Figure 8). Despite this prolonged course of COVID-19 and only once having a negative COVID-19 test, it was initially thought to be new COVID-19 infections with different degrees of severity. It was at this point that the patient’s respiratory symptoms were considered to be compatible with interstitial lung disease, and the patient was considered to have pauci-symptomatic COVID-19.

**Figure 9:**
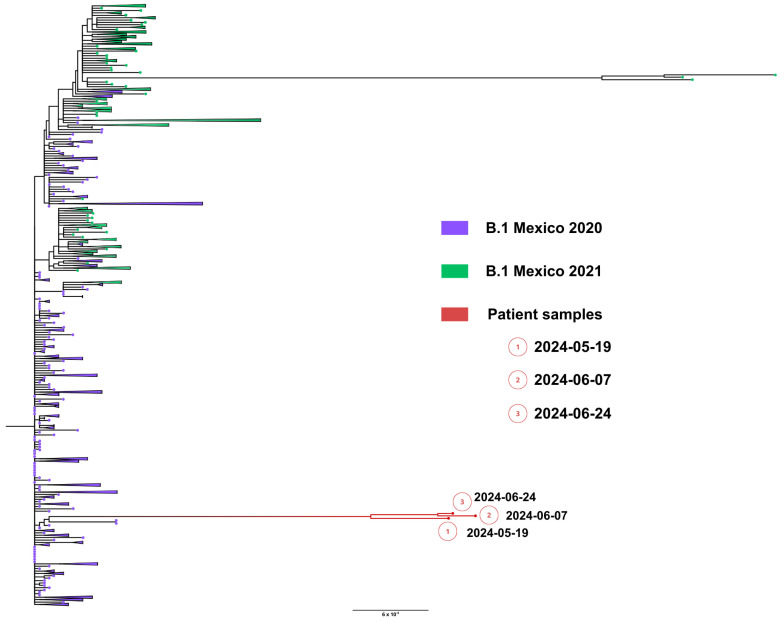
Maximum likelihood phylogenetic tree constructed from whole-genome consensus SARS-CoV-2 sequences, including three longitudinal samples obtained from Case 3 (red tips) and 234 contemporaneous sequences of lineage B.1 from Mexico during 2020–2021. Case 3-derived sequences form a well-supported monophyletic cluster, consistent with persistent infection and intra-host viral evolution. Bootstrap values (>90%, 1000 replicates) support the clustering of patient-derived sequences. The scale bar indicates nucleotide substitutions per site.

Whole genome sequences were obtained from samples from nasopharyngeal swabs from 19 May (1449 days), 7 June (1466 days) and 24 June (1490 days), 2024. All sequenced SARS-CoV-2 genomes showed a unique lineage B.1, confirming persistent infection. Remarkably, the lineage B.1 preceded any variant of concern (VOC) and was prevalent during all of 2020. Phylogenetic analysis showed that viral genomes formed a robust monophyletic clade (Figure 9), consistent with persistent infection and intra-patient viral evolution. Patient sequences clustered with sequences circulating in Mexico in 2020 but were highly divergent to the rest of the isolates and patient sequences (Figure 9).

**Figure 10:**
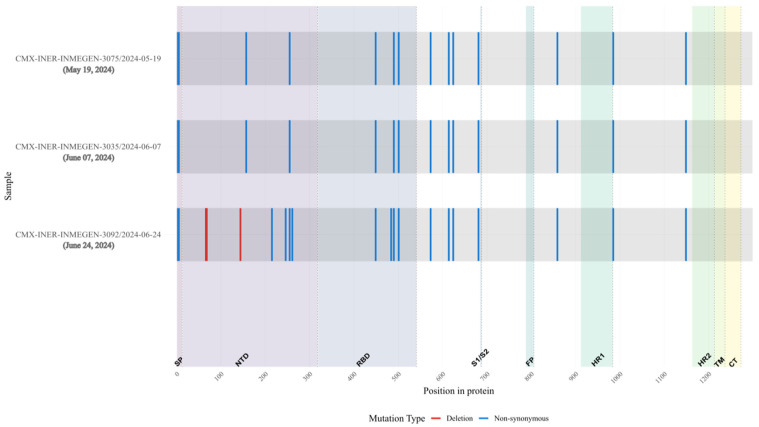
Overview of substitutions present in the Spike protein genome over the course of infection, Case 3. Consensus mutations in the 3 samples sequenced. All nucleotide substitutions in Spike are shown. Amino acid substitutions are indicated for all non-synonymous substitutions (blue). Deletions are shown in red. Spike proteins’ domain positions are indicated.

**Figure 11:**
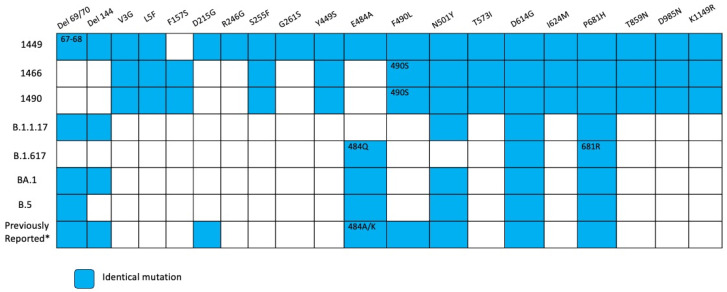
Overview of substitutions present in the Spike protein genome over the course of infection, Case 3. Summary of mutation site shared with variants of concerns such as alpha, delta, omicron BA.1, omicron BA.2, and previous mutations reported in persistent virus. Blue squares indicate the same amino acid position and number inside different amino acid changes. * According reference [9,14].

Compared with lineage B.1, mutations were detected in almost all genes including E, M, N, Spike, Orf1a and Orf1b (Figure 10, Tables S3–S5). Substitutions in Spike detected in several VOCs related with immunological response evasion or transmission increase, like E484A, F490L, N501Y and P681H (Figure 11), were observed in the three sequences of this patient. Also, we detected N-terminal deletions (67–68) in the first sample. These substitutions are shared with persistent virus reported in other immunocompromised patients (Figure 11).

## 4. Discussion

In this study we present three cases of prolonged COVID-19 in three patients with severe immunodeficiency, one with rituximab-treated follicular lymphoma, a second with advanced HIV infection in a patient who abandoned antiretroviral therapy five years before being diagnosed with severe COVID-19, and a third a patient with a mediastinal mass diagnosticated concomitantly with COVID-19, which later turned to be a Thymoma; all these three cases share prolonged viral shedding with different patterns of gene mutations.

Following the acute phase of SARS-CoV-2 infection, various outcomes can be observed, including recovery; death; long COVID-19, marked by prolonged symptoms; and persistent COVID-19. In cases of persistent COVID-19 individuals, SARS-CoV-2 continues to replicate, often shown with the detection of subgenomic RNA or the isolation of the same initial variant, sometimes beyond the respiratory tract [15]. The persistence of an RNA virus is inherently associated with mutations and intra-host viral evolution, which gives rise to highly mutated variants. This phenomenon appears to be relevant when such variants are transmitted and repeated, generating highly mutated and transmissible variants [16].

However, formal definitions for persistent COVID-19 are still lacking, complicating the clinical landscape and the identification of reliable markers to confirm infection resolution. Subgenomic RNA has been found in about 15% of tissue samples up to two months post-infection [17], suggesting the potential for viral persistence in tissues that are less accessible to conventional swab sampling [17].

Some individuals may remain asymptomatic for months, exhibiting persistent viral RNA or infectious virus without respiratory symptoms or imaging abnormalities [18,19,20]. However, immune-cell activation profiles in some individuals may show changes. In other cases, there is imaging or histopathological evidence of organ damage such as interstitial pneumonia, fibrosis, and vascular thrombosis, despite negative nasopharyngeal RT-PCR results. In such cases, viral RNA has been detected in various tissues, corresponding to symptoms of long COVID-19 [17]. Some patients may experience episodes of viral resurgence after initial resolution, with renewed RT-PCR positivity and even reappearance of symptoms or elevated viral loads [21,22,23]. This phenomenon may result from incomplete viral clearance or fluctuating immune responses influenced by underlying health conditions or treatments, which allow the virus to re-emerge in detectable quantities.

In the present study, all patients were over 50 years old. Cases 1 and 3 involved female patients: one with lymphoma who became RT-PCR negative 290 days after initial detection, and another with thymoma and Good’s Syndrome, who retained a Wuhan-like virus strain for over four years (1600 days). Notably, neither of these two cases showed significant mutations in the S-coding region or any mutations commonly associated with “chronic” infections [14]. The remaining case involved a male patient living with HIV with advanced disease, who remained RT-PCR positive for 315 days. In contrast to the other cases, this patient’s viral genome displayed multiple mutations in the S gene and several other coding regions, many of which have been reported in chronic infections.

Immunocompromised patients with different spectrums of immunosuppression often exhibit extended periods of viral shedding, averaging five times longer than immunocompetent individuals [24]. Patients receiving anti-CD20 therapies (e.g., rituximab) for autoimmune diseases, including rheumatoid arthritis, multiple sclerosis or CD20+ lymphomas, not only have more severe COVID-19 outcomes but also an increased risk of prolonged RT-PCR positivity and extended symptoms, likely due to reduced B-cell counts, hypogammaglobulinemia or impaired neutralizing antibody production [25,26,27,28,29,30,31,32]. Patients with B-cell dysfunction tend to experience longer persistence than those with other types of immunosuppression, including hematopoietic stem-cell or solid organ transplantation, advanced HIV disease, and autoimmune illness. However, this varies across studies due to the complexity of cohorts and treatments [15]. A prospective Italian study, not stratified for drug therapy or B-cell alterations identification, found that autoimmune diseases, chronic kidney disease, and medium-high viral load were associated with virus persistence [33].

COVID-19 patients with Good’s Syndrome after thymoma removal have been reported before, and there is evidence that they can have infections persisting for months or years; unfortunately, not all cases have detailed molecular biology follow-up [34,35,36,37]. Case 3, reported here, remained positive after years with a highly mutated variant with no mutations in S, suggesting that immunological deficiencies may have a limited impact and the other factors such as the appearance of fitness mutations, which may be more relevant.

Solid organ transplant recipients, for example, may display delayed antibody production—especially of anti-S IgG and IgA—and a faster decline in antibody levels relative to immunocompetent patients. Persistent infections in these individuals are often characterized by a high CD4+ to CD8+ T-cell ratio, suggesting an altered immune profile [4,16]. Prolonged immune activation can contribute to T-cell exhaustion, potentially diminishing viral clearance ability and fostering conditions for persistence.

Related with severe disease and persistent infection, although at various moments patients had hospitalization due to COVID-19 and oxygen requirements, it is important to mention that all patients had comorbidities that made them susceptible to other infections. In none of the cases was persistent COVID-19 considered as an initial diagnosis because of the time elapsed, but it was not until whole-genome sequencing that, retrospectively, these patients were found to have persistent COVID-19, with the respiratory insufficiency caused by other factors.

Some mutations are associated with immune escape from neutralizing or monoclonal antibodies or to changes in virus fitness, which may vary across variants of concern [12]. Mutations in key regions, such as the S receptor-binding domain, have been associated with enhanced immune evasion. For instance, E484K is a notable mutation that helps the virus escape antibody-mediated neutralization. Some cases with minimal immune pressure, such as those lacking neutralizing antibodies, show fewer mutations, suggesting that immune interaction drives viral evolution. Various cases of persistent COVID-19 lasting around 500 days or more have been reported. In all cases, these are immunocompromised or oncological patients with a complicated SARS-CoV-2 treatment history [19,27,38]. Viral genome sequencing has revealed mutations in the region coding for the Spike protein and elsewhere from the original infection [8,9,10]. In this report, two patients (person living with HIV, and patient with thymoma) had mutations in the S protein, which might be related to immunological escape variants.

There were two kinds of substitutions in two patients in our study, hallmark mutations of VOCs (positions 484, 501, 681 and deletions in the N-terminal region) and substitutions reported in other immunocompromised patients [9]. The presence of substitutions that are characteristics of different VOCs is compatible with the hypothesis of the convergent evolution between viruses in immunocompromised patients and VOCs. Also, there were observed substitutions reported in other immunocompromised patients specifically in positions 27, 95, 183, 215, 340, 346, 356, 417, 440, 446, 478, 484, 486, 490, 493, 501, 614, 681 and deletions in N-terminal domain 63–70 and 142–144. Although some mutations are shared between the two sets of virus, the difference could be explained by evolutionary process within a chronic patient compared with evolution due to transmission in the general population [8].

SARS-CoV-2 is not unique in its persistence ability; other viruses, including respiratory viruses, Zika [39,40] and Ebola [41,42,43], have been found to have extended persistence, often attributed to their ability to reside in tissue reservoirs. Furthermore, superinfections [44] can complicate the scenario and promote further genomic diversity by generation of mutated recombinants [45].

Two unique deletions in N and ORF7b/ORF8 and mutations in Spike, ORF1a, ORF1b, and N were detected. Non-synonymous mutations were found mainly in Spike proteins and one region with temporary substitutions and deletions in ORF1b and other proteins. The omicron virus has undergone non-synonymous mutations, indicating that it developed under positive immune pressure [1]. The possibility of this leading to new variants in communities is concerning [13].

This study has several limitations. First, the number of cases is small and heterogeneous, reflecting the rarity and clinical diversity of prolonged SARS-CoV-2 infection in profoundly immunocompromised individuals. Second, although longitudinal whole-genome sequencing allowed the documentation of within-host viral evolution over extended periods, deep quasispecies analysis based on high-depth amplicon sequencing was not available, limiting fine-scale resolution of minority variants and precluding definitive inference regarding selective pressures. Third, viral isolation and in vitro characterization could not be performed for all cases, and surrogate markers of ongoing replication (e.g., subgenomic RNA) were used when appropriate. Finally, given the observational nature of the study and the complexity of the host immune system and therapeutic interventions, causal relationships between immune status, antiviral treatment, and specific viral evolutionary patterns cannot be established. Accordingly, our findings are intended to provide descriptive longitudinal insights into SARS-CoV-2 persistence rather than mechanistic conclusions.

The current evidence suggests the importance of optimal antiviral interventions, such as with remdesivir and ensitrelvir [46,47], to mitigate the risk of persistent infection in immunodeficient patients. Suboptimal antiviral treatment may lead to incomplete viral clearance and extended viral replication. Further research is required to establish best practices for preventing virus persistence in patients with prolonged viral shedding and to develop strategies to boost immune responses in immunocompromised populations ahead of vaccinations and prevent severe infections.

## Figures and Tables

**Table 1:** Polymorphisms and deletions in persistent replicative SARS-CoV-2 of rituximab-treated patient (day 208).

Polymorphism Position	Nucleotide Change	Amino Acid Change	Protein
758	C/T	H/H	NSP1
771	T/C	V/A	NSP1
1119	A/G	G/R	NSP2
1125	G/A	K/K	NSP2
2037	C/T	A/V	NSP2
2980	G/T	D/G	NSP2
2796	A/G	M/I	NSP2
4891	C/T	T/T	NSP3
Deletion	6554–6776	2097–2171	NSP3
7677	G/A	K/R	NSP3
8653	G/T	M/I	NSP4
9204	A/G	D/G	NSP4
13,131	A/C	Q/P	NSP10
14,408	T/C	L/P	NSP12
17,589	T/C	T/T	NSP13
Deletion	29,513–29,903	414–420	N

## Data Availability

The genomic information generated during the current study is available in the GISAID database. [GISAID] [https://gisaid.org/] [EPI_ISL_15288392, EPI_ISL_17960444, EPI_ISL_17960440, EPI_ISL_17960441, EPI_ISL_17960442, EPI_ISL_17960443 and EPI_ISL_18059424].

## Supplementary Materials

The following supporting information can be downloaded at: https://www.mdpi.com/article/10.3390/v18020189/s1, Table S1. Identified mutations and deletions in persistent replicative SARS-CoV-2 of person living with HIV (Case 2). N, ORF1a, ORF3a and ORF9b genes. Table S2. Identified mutations and deletions in persistent replicative SARS-CoV-2 of person living with HIV (Case 2), Spike gene. Table S3. Identified mutations, deletions and frame shift in persistent replicative SARS-CoV-2 of thymoma patient (Case 3). E, M, N, ORF3a, Orf7a, Orf8 and ORF9b genes. Table S4. Identified mutations in persistent replicative SARS-CoV-2 of thymoma patient (Case 3). ORF1a and Orf1b genes. Table S5. Identified mutations and deletions in persistent replicative SARS-CoV-2 of thymoma patient (Case 3). ORF6, Orf7b, Orf8 and Spike genes.
