# Supplementary material for: Molecular Characterization of Persistent SARS-CoV-2 Infections in Immunocompromised Patients
Source: Viruses. 2026 Jan 30;18(2):189. doi: 10.3390/v18020189 (PMC12944910; doi:10.3390/v18020189)
Supplement: Supplementary file 1 [file viruses-18-00189-s001.zip › viruses-4062267-supplementary.pdf]

Table S1. Identified mutations and deletions in persistent replicative SARS-CoV-2 of person living with HIV (Case 2). N, ORF1a, ORF3a and ORF9b genes.

	Identified Mutations			
**Day of sample (GISAID code)**	**N**	**ORF1a**	**ORF3a**	**ORF9b**
Day 0 (CMX-INER-IBT-2627/2023-07-13)	E31-, R32-, S33-	F1632I, P1640L, P2018L, L3201F, T4065I, S3675-, G3676-, F3677-	S74F	E27-, N28-, A29-
Day 8 (CMX-INER-IBT-2649/2023-08-04)	E31-, R32-, S33-	I16321F, P1640L, P2018L, S2024L, L3201F, E3962K, S3675-, G3676-, F3677-	S74F	E27-, N28-, A29-
Day 140 (CMX-INER-2760/2023-11-30)	E31-, R32-, S33-	P1640L, P2018L, S2024L, L3201F, E3962K, S3675-, G3676-, F3677-	S74F	E27-, N28-, A29-
Day 181 (CMX-INER-2790/2024-01-10)	E31-, R32-, S33-	H1500Y, P1640L, P2018L, S2024L, T2408A, L3201F, E3962K, S3675-, G3676-, F3677-	S74F	E27-, N28-, A29-
Day 189 (CMX-INER-INMEGEN-2812/2024-01-18)	E31-, R32-, S33-	A498V, P1497S, P1640L, P2018L, S2024L, L3201F, E3962K, P4337S, S3675-, G3676-, F3677-	S74F	E27-, N28-, A29-
Day 314 (CMX-INER-INMEGEN-3017/2024-05-22)	D144H, E31-, R32-, S33-	H1500Y, P1640L, P2018L, S2024L, G2265V, L3201F, E3962K, P4337S, S3675-, G3676-, F3677-	A39D, S74F, H78Y, V256-, N257-	E27-, N28-, A29-

Marked in green are those mutations associated with virus persistence (as listed in harari 2024)

Table S2. Identified mutations and deletions in persistent replicative SARS-CoV-2 of person living with HIV (Case 2), Spike gene.

**Sample**	**Mutaciones proteina S**
CMX-INER-IBT-2627/2023-07-13	P9L,A27S,K77E,T95I,R158K,Q183E,G213E,D215G,D339G,R346T,G446S,L452R,A484K,F486L,R493Q,P621S,H681Y,L841R,D936Y,D1146N,L24-,P25-,P26-,C136-,N137-,D138-,P139-,F140-,L141-,D142-,V143-,Y144-
CMX-INER-IBT-2649/2023-08-04	P9L,A27S,H69D,T95I,R158K,Q183E,G213E,D215G,R346T,G446S,L452R,A484E,F486L,R493Q,V615A,V642G,L841R,D936H,L24-,P25-,P26-,C136-,N137-,D138-,P139-,F140-,L141-,D142-,V143-,Y144-
CMX-INER-2760/2023-11-30	P9L,A27S,K77E,T95I,R158K,Q183E,G213E,D215G,R346T,R403K,N417T,G446N,L452R,A484K,F486L,R493Q,P621S,H681Y,L841R,D936Y,D1146N,L24-,P25-,P26-,C136-,N137-,D138-,P139-,F140-,L141-,D142-,V143-,Y144-
CMX-INER-2790/2024-01-10	P9L,A27S,K77E,T95I,R158K,Q183E,G213E,D215G,R346T,V367L,R403K,N417T,G446N,L452R,V483L,A484K,F486L,R493Q,V615A,V642G,L841R,D936H,D1146N,L24-,P25-,P26-,C136-,N137-,D138-,P139-,F140-,L141-,D142-,V143-,Y144-
CMX-INER-INMEGEN-2812/2024-01-18	P9L,A27S,H69D,T95I,R158K,Q183E,G213E,D215G,R346T,N417T,G446N,L452R,A484Q,F486L,R493Q,H505Y,P621S,H681Y,D936Y,D1146N,L24-,P25-,P26-,C136-,N137-,D138-,P139-,F140-,L141-,D142-,V143-,Y144-
CMX-INER-INMEGEN-3017/2024-05-22	P9L,A27S,K77E,T95I,M153L,R158K,Q183E,G213E,D215G,I332V,E340K,R346T,K356T,R403K,N417T,K440R,K444R,L452R,K478I,F486P,R493Q,S494P,G496S,V615A,V642G,H681R,A688V,G838S,L841R,D936H,S939F,E1150K,L24-,P25-,P26-,C136-,N137-,D138-,P139-,F140-,L141-,D142-,V143-,Y144-,Y145-,H146-,K147-,N148-,N149-,K150-,S151-,W152-,L244-,H245-,R246-,S247-,Y248-,L249-,T250-,P251-,G252-,D253-,S254-,S255-,A484-

Residues marked in yellow/green are those identified as recurrent mutations in putative chronic infections and in many cases, for example mutation R346T identified as an escape mutation, that change virus fitness (Harari et al., 2024)

Table S3. Identified mutations, deletions and frame shift in persistent replicative SARS-CoV-2 of thymoma patient (Case 3). E, M, N, ORF3a, Orf7a, Orf8 and ORF9b genes.

seqName	frameShifts	aaSubstitutions E, M, N, Orf3a, Orf7a, Orf8 and Orf9b genes
CMX-INER-INMEGEN-3075/2024-05-19	ORF6:2-62,ORF7b:14-44	E:L21F,M:S197T,N:D22Y,N:S194L,N:G238C,N:Q408L, ORF3a:Q38E,ORF3a:G49C,ORF3a:D155Y,ORF7a:S37F,ORF7a:T39I,ORF7a:A105S,ORF7a:E121*,ORF8:Q18*,ORF8:A65S,ORF8:F120V,ORF8:I121L,ORF9b:Q18H
CMX-INER-INMEGEN-3035/2024-06-07	ORF6:2-40,ORF8:68-122	E:L21F,M:I82T,M:S197T,N:D22Y,N:S194L,N:G238C,N:Q408L, ORF3a:G49C,ORF3a:D155Y,ORF7a:E22D,ORF7a:S37F,ORF7a:A105S,ORF7a:E121*,ORF7b:I2T,ORF8:Q18*,ORF9b:Q18H
CMX-INER-INMEGEN-3092/2024-06-24	ORF6:2-40,ORF8:68-122	E:L21F,M:I82T,M:S197T,N:D22Y,N:S194L,N:G238C,N:Q408L,ORF3a:G49C,ORF3a:D155Y,ORF7a:E22D,ORF7a:S37F,ORF7a:A105S,ORF7a:E121*,ORF8:Q18*, ORF9b:Q18H

Table S4. Identified mutations in persistent replicative SARS-CoV-2 of thymoma patient (Case 3). ORF1a and Orf1b genes.

seqName	aaSubstitutions Orf1a	aaSubstitutions Orf1b
CMX-INER-INMEGEN-3075/2024-05-19	V86F,S166G,T1558K,I1568V,T1637I,T1682I,S1856F,S2024L,A2098T,T2158I,T2274I,N2361K,S2900L,E2904D,P3359S,K3577N, L3606F,V3917G,V4102I	P314L,N480T,P1325S,R2000H,V2178F
CMX-INER-INMEGEN-3035/2024-06-07	V86F,S166G,P309L,D992Y,E1564Q,T1637I,T1682I,S2024L,A2098T,T2158I,R2184I,T2274I,N2361K,S2900L,E2904D,T3287I,P3359S,K3577N,L3606F,A3889V,V4102I	P314L,N480T,P1325S,R2000H,V2178F
CMX-INER-INMEGEN-3092/2024-06-24	V86F,S166G,P309L,D992Y,T1558K,E1564Q,T1637I,T1682I,S2024L,A2098T,T2158I,R2184I,T2274I,N2361K,S2900L,E2904D,P3359S,K3577N,T3579I,L3606F,K3886R,V4102I	P314L,N480T,P1325S,R2000H,V2178F

Table S5. Identified mutations and deletions in persistent replicative SARS-CoV-2 of thymoma patient (Case 3). ORF6, Orf7b, Orf8 and Spike genes.

seqName	aaSubstitutions Spike	aaDeletions
CMX-INER-INMEGEN-3075/2024-05-19	V3G,L5F,D215G,R246G,S255F,G261S,Y449S,E484A,F490L,N501Y,T573I,D614G,I624M,P681H,T859N,D985N,K1149R	ORF6:M1-,ORF7b:F13-,ORF8:I58-,S:H66-,S:A67-,S:I68-,S:Y144-
CMX-INER-INMEGEN-3035/2024-06-07	V3G,L5F,F157S,S255F,Y449S,F490S,N501Y,T573I,D614G,I624M,P681H,T859N,D985N,K1149R	ORF6:M1-,ORF6:S41-,ORF8:I58-,ORF8:A65-,ORF8:G66-,ORF8:S67-
CMX-INER-INMEGEN-3092/2024-06-24	V3G,L5F,F157S,S255F,Y449S,F490S,N501Y,T573I,D614G,I624M,P681H,T859N,D985N,K1149R	ORF6:M1-,ORF6:S41-,ORF8:I58-,ORF8:A65-,ORF8:G66-,ORF8:S67-
